# The Perceived Social Determinants of Mental Health among African Youth Refugees in South Australia

**DOI:** 10.1007/s10903-025-01728-4

**Published:** 2025-07-05

**Authors:** William Mude, Patrice Whitehorne-Smith, Tafadzwa Nyanhanda, Lillian Mwanri

**Affiliations:** 1 Flinders University, Adelaide, Australia; 2 Victoria University, Melbourne, Australia; 3 Torrens University Australia, Adelaide, Australia

**Keywords:** Determinants, Mental health, Immigrants, Adolescents, South Australia

## Abstract

This qualitative study explored the social determinants of mental health among African youths in South Australia, revealing five major themes: displacement and migration, family relationships and dynamics, social exclusion, racism and discrimination, and unemployment and education. The findings indicate that many participants experienced significant displacement due to armed conflict, leading to prolonged migration journeys and feelings of disconnection and instability upon their arrival in Australia. Cultural tensions arose as participants grappled with reconciling their heritage with Australian culture, often resulting in mental health challenges and harmful behaviours. Furthermore, while family connections provided important emotional support, many youths faced emotional burdens due to separations from their families and changes in family dynamics. The study also addresses the impact of racism and discrimination on their experiences, showing how stereotypes and prejudices further isolate young people and hinder their meaningful participation in society. These findings, therefore, highlight the urgent need for additional support systems to foster community connections and promote mental well-being among youths from refugee backgrounds.

## Background

In mid-2024, the United Nations High Commission for Refugees (UNHCR) reported that 122.6 million people worldwide, including 47 million children, were forcibly displaced due to persecution, conflict, violence, human rights violations, or other serious disruptions to public order [1]. Among these, over 37.9 million were classified as refugees, with an additional eight million identified as asylum seekers [1]. Many of these individuals endure traumatic experiences such as torture, violence, and loss of family members during their displacements, which can have lasting psychological impacts [2].

After fleeing their homes, refugees often face significant challenges in adapting to new environments, whether in refugee camps or upon resettlement in countries like Australia. Barriers to employment, housing insecurity, limited educational access, language difficulties, financial constraints, and a lack of social support are common hurdles they must navigate [3, 4]. Moreover, ethnic minorities living in a predominant majority culture often face psychological stress and anxiety stemming from the conflict between their cultural identities and the pressures to assimilate, illustrating the complex dynamics of social and ethnic identity and acculturation [5]. In addition, experiences of racism and discrimination can lead to increased stress, anxiety, and depression, significantly affecting migrants’ overall mental health and well-being [6].

Research has explored the connections between resettlement experiences and health outcomes among adult refugees [7, 8], contributing valuable insights to knowledge and policy in this area. However, there is a notable gap in understanding the experiences of youths from refugee backgrounds in Australia and how these experiences intersect with their mental health and well-being. Recognising the social determinants of mental health is vital for unpacking the intersections between the experiences of youths and their mental health outcomes and addressing inequities and disadvantages. Such findings can also be used to design interventions and improve overall health outcomes. For example, research indicates that tailored services, such as peer-led and community-driven initiatives, are important in addressing mental health among this population group [9, 10].

Employing a qualitative research approach, this study investigates the experiences of African youths from refugee backgrounds in South Australia, aiming to illuminate how these experiences determine their overall mental health outcomes. Approximately 20,000 people of African backgrounds lived in South Australia in 2021, and a significant proportion of those lived in the Adelaide metropolitan area [11].

### Theoretical Framework

This study is grounded in the social determinants of mental health framework, which the World Health Organization and Calouste Gulbenkian Foundation [12] defined as the conditions influencing individuals’ everyday life throughout the life course, starting with life prior to birth and extending through early childhood, adolescence, family-building and working ages, and into older age. These determinants include individual factors like age and education, lifestyle choices, social networks, and living and working conditions. They also encompass broader socioeconomic, cultural, and environmental influences, such as immigration policies.

Therefore, the World Health Organization has developed a comprehensive Mental Health Action Plan for 2021–2030, which aimed at improving “more effective leadership and governance for mental health; the provision of comprehensive, integrated mental health and social care services in community-based settings; implementation of strategies for promotion and prevention; and strengthened information systems, evidence and research” [5, 13].

Moreover, while various factors can negatively impact mental health, there are also protective factors that help maintain and enhance it, such as strong social support systems, access to mental health resources, and promoting resilience through education and healthy lifestyle choices [14]. Therefore, this entails strengthening these protective factors for good mental health at the individual, societal, and structural levels, as well as adopting holistic approaches to health promotion [15].

## Methods

### Study Design and Data Collection

This study employed a qualitative research design. It attempted to answer the research questions, “What are the perceived social determinants of mental health among African youth refugees in South Australia, and how do these factors impact them?” Data collection for this study occurred through semi-structured one-on-one interviews and focus group discussions with African youths from refugee backgrounds in Adelaide. The interview questions were developed in consultation with community leaders. The community leaders were recruited through a resettlement services provider in Adelaide. They were part of the study’s steering committee and participated in the study’s design and governance, aligning with the principles of co-production of research [16]. The study was piloted with seven community leaders to assess its cultural appropriateness, as per van Manen [17] Guidelines.

The interviews and focus group discussions took place in a central location frequently used by African youths from refugee backgrounds and were conducted by two investigators. All interviews were conducted in English and asked questions about migration, family, education, work, social networks and support, community, and experiences in Australia. The interviews ended after it reached data saturation, when no new information was forthcoming from the subsequent interviews [18].

### Study Participants

 Purposive sampling was used to identify participants, and they were recruited through a snowballing strategy [19]. Participants provided written informed consent prior to participating in the study, and they received $30 as a token of appreciation. Twenty-three youths participated in this study. Four were female, and 19 were male. The average age of participants was 24. Their length of stay in Australia ranged between 2 and 12 years. The youths came mainly from the Central, Eastern, and Western African regions. The interviews and focus group discussion data were audio recorded using a digital recorder and transcribed verbatim.

### Data Analysis

**Fig. 1:**
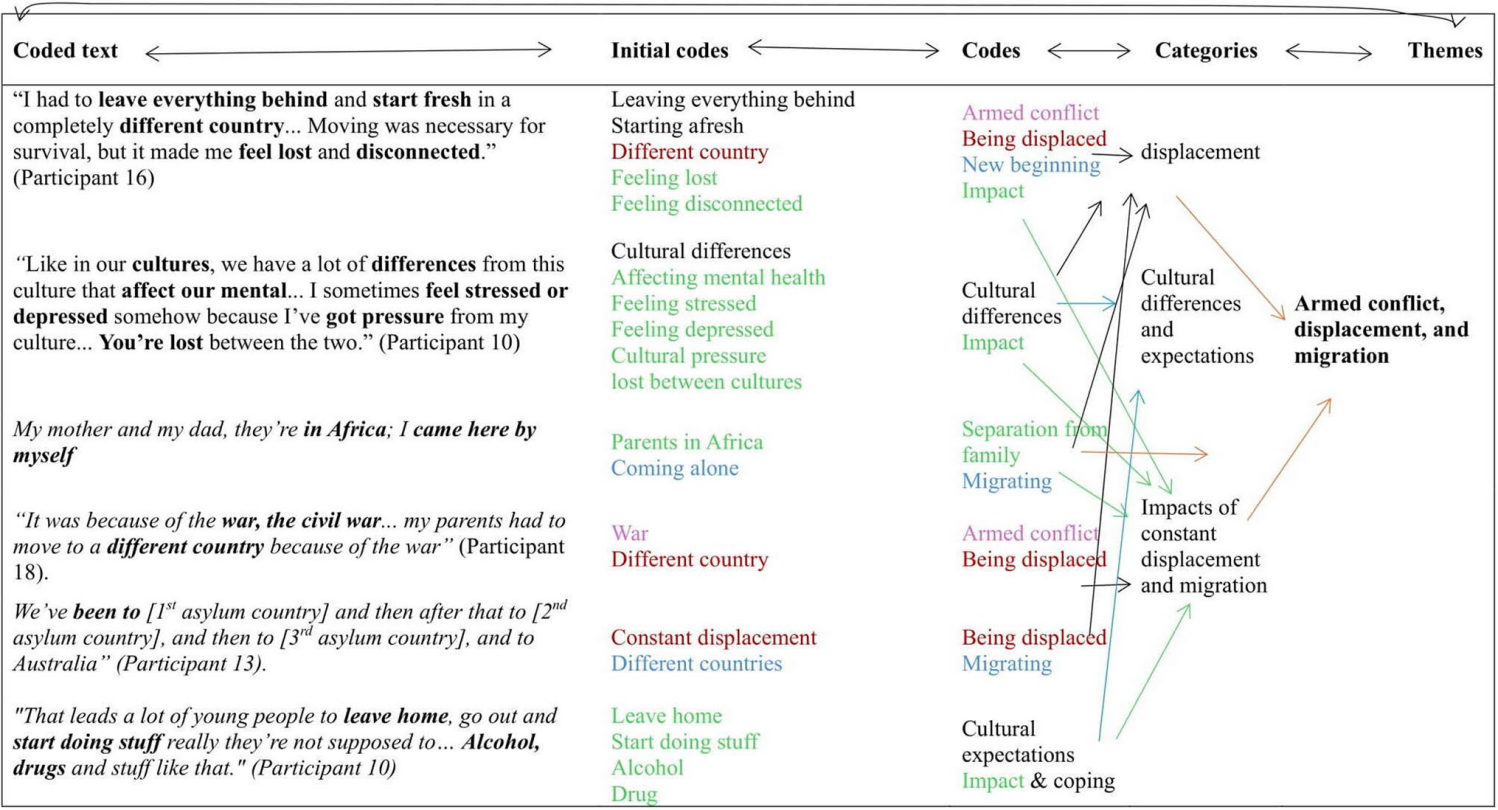
An example showing a process used to generate themes

The interpretive thematic analysis framework of Braun and Clarke [20] was used to analyse the qualitative data. Two investigators analysed the data by reading and re-reading the transcripts to gain insights into the data while using NVivo to code key concepts and meanings [20]. The coded concepts were categorised into themes, and the emerging themes were refined and analysed further by moving back and forth between ‘the parts and the whole’ of the transcripts to identify the main themes, providing deep insights into their determinants of mental health outcomes. All themes were reviewed, analysed, and linked to the research question and the social determinants of mental health. A diagrammatic example showing the process of generating the themes is presented in Fig. 1 below.

Ethical Approval for the study was sought from the Flinders University Social and Behavioural Research Ethics Committee (SBREC project number 5480).

## Findings

Five major themes emerged from this study regarding the perceived social determinants of mental health among African youths in South Australia. These themes included displacement and migration, family relationships and dynamics, social exclusion, racism and discrimination, and unemployment and education.

### Displacement and Migration

In this study, many young people revealed experiencing displacement and migration across multiple countries because of armed conflicts before coming to Australia. One participant noted, *“We have been to [1st asylum country] and then after that to [2nd asylum country]*,* and then to [3rd asylum country]*,* and to Australia”* (Participant 13). Often, these journeys involved long durations in another country before arriving in Australia, creating a feeling of temporality or living in suspension. For example, a participant demonstrates this sentiment of experiencing long durations in the migration process before reaching Australia by noting, *“I was in [asylum country] throughout… It is too long; I have been there right through”* (Participant 4).

Some participants recounted their experience of being uprooted from their familiar environment and moving to a foreign country to start their lives afresh from scratch. Although this was important for their safety and security, participants struggled to connect and form their identities. As a participant noted, due to armed conflict, “*I had to leave everything behind and start fresh in a completely different country… Moving was necessary for survival*,* but it made me feel lost and disconnected”* (Participant 16). This statement reflects the instability and uncertainty young individuals experienced before reaching Australia, which could profoundly affect their sense of security and belonging in Australia. The young people in this study experienced family separation during their migration, they migrated to Australia alone or with an older member of their extended family. For example, a participant described this sentiment, *“My mother and my dad*,* they are in Africa; I came here by myself”* (Participant 11).

Additionally, migrating to Australia created different kinds of cultural tensions. Young people experienced pressure to reconcile their Australian and heritage cultures. On the one hand, they felt pressure to uphold their culture; on the other, they felt pressure to belong to Australian culture. This experience created a sense of being torn between Australian culture and their heritage, resulting in confusion and stress. For example, a participant states, *“Like in our cultures*,* we have many differences from this culture that affect our mental… I sometimes feel stressed or depressed because of pressure from my culture… You are lost between the two”* (Participant 10).

Based on the narrative of the young people who participated in this study, conflicting cultural expectations caused some young people to engage in harmful behaviours as a form of coping. Migration-induced cultural differences between young people and their parents or guardians have created significant family tension, as parents try to maintain traditional authority while young people seek independence within the Australian culture. Experiences of family loss and grief often exacerbate this conflict. For instance, one participant stated, *“My father is not here… He is late now; he died. If something happens and I want to talk*,* my mother shouts*,* and I have to shut up”* (Participant 18). As noted by another participant, such struggles can lead young people to leave home for independence: *“I am with my aunt here*,* and we have a lot of disagreements… that is what leads many young people to leave home*,* go out and start doing stuff they are not supposed to… Alcohol*,* drugs and stuff like that”* (Participant 10). This tension can result in negative coping strategies, including substance use and overeating, as individuals seek to manage their stress and emotions, with one participant revealing, *“When I am home*,* all of this stuff comes into my head… Sometimes I just gain weight because I am stressed”* (Participant 13). Another participant stated, “*They struggle with the family*,* but most of them do not talk about it… they just keep it to themselves and drink a lot”* (Participant 22).

### Family Relationships and Dynamics

This study found that seeking emotional support remained challenging, especially when many young people are separated from their families and have had to depend on long-distance communication. One participant observed, *“I have my mum in Africa… I always call her when I am stressed and just talk to her on the phone for hours”* (Participant 13). Additionally, family separations led to emotional burdens among young people and a feeling of powerlessness to change the situation. A participant noted, *“Being away from family makes everything harder; you feel like a piece of you is missing…sometimes I wish I could just go home*,* but the distance makes that impossible”* (Participant 16).

Changes in family responsibilities and structures following migration to Australia, leading to life pressure, were expressed. Many young people described their experience of feeling pressured to provide financial support to their families, who were left in refugee camps or their country of origin. One participant stated, *“… the pressure behind - back in [name of a country] you have to support your family”* (Participant 10). Moreover, young people felt obligated to provide support, with one participant noting, *“All my money goes home”* (Participant 1). Another participant added, *“You see*,* I am happy*,* but inside*,* I am not happy because… our mum is in Africa… I have eight people*,* a father and three sisters in [name of a city in Africa]”* (Participant 4). Similarly, young people felt their families expected them to get employment to support the family members left behind, with a participant stating, “*my family would say ‘okay*,* he is in Australia*,* and he is going to have money*,* he will get a job and will support us’”* (Participant 11).

### Social Exclusion

Participants in this study revealed experiencing social exclusion. Young people who participated in this study felt isolated and lacked social networks in the community. This lack of community created a sense of isolation and increased the challenges of navigating life in Australia. The following statement captures these sentiments, with one participant stating, *“I do not have my community here*,* like tribe… so for me*,* I do not have that community here”* (Participant 13). Another participant added that, generally, many young people *“do not have that close relationship with family or community to talk to*,* and they keep things to themselves*,* that is where their big problem [mental health] comes”* (Participant 10). These sentiments demonstrate that a lack of community and family support networks could contribute to the mental health struggles of young people.

Moreover, participants in this study expressed several challenges in developing social networks and support. As a result, many participants felt pressured to fit in and form their social networks and support. One participant observed, *“I feel pressure to fit in*,* but it is difficult when your background is so different”* (Participant 16). Another participant added, *“Everyone is feeling it [pressure to fit in]”* (Participant 11), suggesting their collective awareness of the challenge and their solidarity with each other on this issue.

However, in the process of wanting to ‘fit in’, some young people experienced issues of peer pressure, such as needing to engage in negative behaviours, including but not limited to experimenting with substance use, alcohol drinking, and anti-social behaviour. One participant described observing one young person experiencing such peer pressure by noting, *“He was a friend to some guys. When they go for the beach*,* those guys are taking drugs*,* they are drug addicted*,* like heroin or something*,* so those guys gave him drugs”* (Participant 22).

### Racism and Discrimination

Participants in this study described experiencing pervasive racism and discrimination issues. According to the youths, while some young people found ways of dealing with such racism, others were unable, leading them to “take it personal”. A participant demonstrated this sentiment by noting, *“Sometimes when we are together some people behave racist to us*,* but we do not take it personal. Some of my friends take it personal. Yeah*,* I have faced racism which I know is everywhere but sometimes I think it is too much in Australia”* (Respondent 4).

There was a sense among young people that they were being stereotyped and unfairly targeted by law enforcement agencies. One participant observed, *“Police are not getting along with us. If they see us*,* you know*,* if you just drive in the street*,* they are going to stop you in the street ‘where are you going? Why are you driving this?’”* (Participant 7). Some participants revealed that law enforcement officers do not allow them to discuss their rights. For example, one participant stated, *“When you try to tell your rights to the police*,* police refuse unless you accept what they tell you”* (Participant 21).

Participants also talked about the media portrayal of them to the public without thinking about the negative implications that such views create in the public. Participants felt that the media intentionally portrays them negatively and names their communities to stereotype them. They said the naming of communities when media report on a case has led to a negative view of them in the public and to being unfairly stereotyped. One participant noted, *“‘oh [two community names] youths have been doing crimes*,* [crimes] increased compared to the other communities’… If you are from that community people think like ‘oh he is probably one of those people’”* (Participant 21).

Participants reported experiences of discrimination, emphasising the subtle yet pervasive racism that affects job opportunities and self-esteem. One individual noted the challenge of obtaining a job, remarking, *“Normally*,* it is hard to get a job because they say that Australia does not have discrimination*,* but when interviewing alongside white candidates*,* it feels different”* (Participant 11). Another participant shared a troubling encounter with police who fined them for ‘jaywalking’ while observing a group of white individuals pass a red light without consequence. *“They said*,* ‘you guys did jaywalk*,*’ but there was no traffic. I feel like the police are targeting us*,* not everyone”* (Participant 20).

### Unemployment and Education

Many young people in this study described a substantial challenge they experienced while attempting to gain employment, and the profound impact of the persistent failure and rejection on their mental health, leading some young people to consider self-harm as an escape. For example, one participant demonstrated this sentiment: *“It is the frustration of how you are getting a job… It is troublesome because it has taken me a while. I have been trying to find a part-time job and will get a job where I can support myself. You can apply 1000*,* and then you will be rejected. This comes into the minds of the young people and takes their lives easily”* (Participant 11).

The impact of not being able to find employment created a sense of frustration among young people. Such frustrations led to a sense of hopelessness and suicidal ideation among young people, with a participant commenting, *“They [youths] do not have a job; they do not go to school… You know*,* they get bad treatment… You know*,* they feel helpless*,* like no one cares about them. They cannot get help*,* and they-you know*,* some of them take their own lives”* (Participant 20).

Although the sentiment around suicidal ideation was common, some participants understood that their situation would change for the better despite the level of financial hardship and poverty they experienced, with one participant stating, *“If you are still alive you are rich because one day you might become rich*,* but if you are dead*,* even though you are rich*,* nobody will call you as rich because you are a dead person”* (Participant 11). This statement demonstrates resilience and emotional strength despite their challenges.

Many youths expressed concerns about their job readiness, highlighting a lack of necessary education and experience. Even after job readiness training, they struggled to find work. One participant recounted their frustration: after multiple failed job applications, they were advised to obtain a certificate to improve their prospects. However, even with the certificate, they could not secure a job due to a requirement for a driver’s license. *“When I came to Australia*,* I was told to do a course for qualification*,* and now that I have the certificate*,* everyone says I lack job experience… I do not have a driver’s license*,* so I cannot get a job. It is frustrating”* (Participant 4).

Such unemployment created financial issues and a sense of failure in young people’s lives, as demonstrated in the statement, *“If you do not get a job*,* you are not in school*,* so you cannot make it in society”* (Participant 3). Many participants described a lack of access to employment opportunities, compounded by a lack of language; they felt disillusioned. For example, a participant stated, *“Young people in our community have no access to some jobs.… Also*,* because we migrants*,* we come– some of us do not know English… they do not have a job*,* so what they decided is they thought they are hopeless”* (Participant 5).

## Discussion

This study investigated the perceived determinants of mental health among young African people of refugee backgrounds in South Australia. The study revealed several determinants impacting the mental health of these young people, including conflict-induced displacement and migration stressors, unemployment, education, family relationships, social exclusion, lack of support networks, and experiences of racism and discrimination. It highlights that the ongoing impacts of displacement can disrupt the social and material foundations necessary for these young people to thrive, often resulting in feelings of isolation due to the absence of familiar community structures.

This study found that the experience of displacement and migration has ongoing impacts on young people’s lives, and familial relationships and community support post-migration shape young people’s experiences as they navigate social challenges in their new environment. The lack of a familiar community structure, material and social support, and family cohesion at home made adaptation more challenging as young people felt isolated [21, 22]. Cultural tension has led to stress and “negative thoughts” and made young people use harmful coping strategies. Tensions between Australian and heritage cultures often lead young migrants to struggle with their multiple identities, causing identity confusion and a decline in self-esteem [23]. Implementing cultural empowerment programs can help young migrants embrace their ethnic backgrounds while fostering a positive connection to their Australian identity [24]. Such initiatives, including cultural education and community engagement, can promote inclusivity and pride in diverse identities, ultimately enriching the fabric of Australian society.

Moreover, separations during displacement and migration altered family responsibilities, suggesting that these young people in Australia not only had to worry about sustaining themselves but also about supporting their families back home. Such expectations to support the family put immense pressure on young people to secure employment, and the persistent rejection makes such situations even dire. Family relationships are an important factor in shaping an individual’s social context, as they help people develop a healthy personality and harness social competencies and social adjustment resources [25]. Grevenstein et al. [25] found that individuals with better family relationships experience reduced psychological distress, improved life satisfaction, greater resilience, higher self-esteem, and increased self-efficacy. This study found that young people still relied on their family members who were left behind for emotional support, demonstrating the enduring bonds between families. However, while such connections are important to provide comfort, they also demonstrate the continuing impact of displacement, contributing to an ongoing emotional struggle of being separated from one’s family and the longing for stability and connection.

Social inclusion and strong support networks are vital for mental health and well-being [26]. Individuals with solid external bonds tend to have better health and increased life expectancy [12]. This study found that young people felt socially excluded, lacked support, and struggled with acceptance amidst judgments from peers, families, and the community. Other studies have shown that racism and discrimination play a central role in fostering and maintaining social exclusion for minority groups and lead to poor mental health outcomes [27, 28]. The cycle of racism, discrimination, and social exclusion created anxiety about belonging and self-identity.

Additionally, peer pressure led many young people to engage in harmful activities to fit in, which affected their mental health [29]. The struggle for acceptance, compounded by discrimination, racism and stereotypes, can affect the self-esteem and mental well-being of young people, making it challenging for them to find supportive networks where they can express themselves and find guidance [30]. Moreover, research indicates that policies implemented to foster community bonding through cultural exchange and mentorship programs, alongside robust anti-discrimination campaigns and strengthened legal protections, can help break the cycle of racism, discrimination, and social exclusion affecting young refugees [31].

Employment is a significant determinant of health, with research finding that it is directly correlated with better physical health outcomes [32]. Moreover, unemployment adversely impacts mental and physical health outcomes [32, 33]. Participants in this study described experiencing heightened stress due to struggles with finding employment, navigating educational systems, and managing familial expectations. The pressure to succeed, coupled with encounters of open hostility and bigotry and societal judgment related to unemployment, can create a cycle of heightened anxiety and depression, making it difficult for individuals to establish financial stability and independence [33, 34].

Despite these factors, evidence suggests that interventions and community engagement activities specifically designed for migrants and refugees are crucial in addressing mental health challenges. For example, strengthening support services, developing peer-led initiatives, fostering youth involvement, and enhancing access to educational and employment opportunities are important for creating an inclusive environment that promotes resilience and well-being [35, 36].

### Study Strengths and Limitations

This study is limited by its ineligibility for generalisation. The views expressed in the findings may not apply beyond the studied participants. Therefore, broader conclusions must be avoided. The fact that the youths were recruited mainly from a central location in Adelaide, frequently visited by African youths from refugee backgrounds, and the recruitment through snowballing means that some young people who did not use this central location did not participate in this study, which could have affected the diversity of views presented in this study. However, the study’s strengths lie in its collaborative nature, with community leaders serving as partners and collaborators in the design, implementation, and governance of the study [16].

### Contribution To the Literature

This study contributes to the literature by identifying critical areas that require targeted intervention for youths from refugee backgrounds, emphasising the importance of tailored support services that enhance access to mental health services, educational programs, and employment assistance, all designed to accommodate the diverse experiences of these individuals. Furthermore, the study advocates for programs that facilitate open dialogue, peer-led initiatives, and strong community networks to counteract issues such as family separation, substance abuse and social isolation. Community leaders’ mentoring is essential for guiding youth towards positive pathways, while targeted vocational training and partnerships with local businesses can address unemployment and underemployment.

### Conclusion

This study sheds light on the multifaceted challenges faced by young people of refugee backgrounds and underscores the importance of a holistic approach to their integration and support. The experiences shared by participants reveal significant issues related to family separation, social pressures, unemployment, and mental health, highlighting the urgent need for tailored interventions and community engagement. Stakeholders should strengthen support services, foster youth involvement, and enhance access to educational and employment opportunities to create an inclusive environment that promotes resilience and well-being. Addressing these critical areas will benefit young people of refugee backgrounds and enrich the broader community, contributing to more substantial and more resilient communities from refugee backgrounds.

## Data Availability

No datasets were generated or analysed during the current study.
